# Identification of Novel Insertions and Deletions in Haematopoietic Stem/Progenitor Cells in de novo Myelodysplastic Syndromes

**DOI:** 10.22088/IJMCM.BUMS.10.3.227

**Published:** 2022-01-10

**Authors:** Manoj Bandara, Hemali Goonasekera, Vajira Dissanayake

**Affiliations:** 1 *Department of Pre-Clinical Sciences, Faculty of Medicine, General Sir John Kotelawala Defence University, Rathmalana, Sri Lanka.*; 2 *Human Genetics Unit, Faculty of Medicine, University of Colombo, Colombo 8, Sri Lanka.*

**Keywords:** Insertions and deletions, myelodysplastic syndromes, haematopoietic stem and progenitor cells, next generation sequencing

## Abstract

Myelodysplastic Syndromes (MDS) are *clonal haematological stem cell disorders*. The molecular basis of MDS is heterogeneous and the molecular mechanisms underlying biology of this complex disorder are not fully understood. Genetic variations (GVs) occur in about 90% of patients with MDS. It has been shown that in addition to the single nucleotide variations, insertions and deletions (indels) in the key genes that are known to drive MDS, could also play a role in pathogenesis of MDS. However, only a few genetic studies have analyzed indels in MDS. The present study reports indels of bone marrow (BM) derived CD34^+^ haematopoietic stem/progenitor cells of 20 newly diagnosed *de novo *MDS patients using next generation sequencing.A total of 88 indels (9 insertions and 79 deletions) across 28 genes were observed. The genes that showed more than five indels are *BCOR *(N=6), *RAD21 *(N=6), *TP53 *(N=8), *ASXL1 *(N=9), *TET2 *(N=9) and *BCORL1 *(N=10). Deletion in the *BCORL1 *gene (c.3957_3959delGGA, TGAG>TGAG/T) was the most recurrent deletion and was observed in 4/20 patients. The other recurrent deletions reported were *EZH2 *(W15X, N=2) and *RAD21 *(G274X, N=3). The recurrent insertions were detected in the *FLT3 *(E598DYVDFREYE, N=3) and in the *NPM1 *(L287LCX, N=3) genes. The findings of this study may have a diagnostic, prognostic and a therapeutic value for MDS after validation using a larger cohort.

Myelodysplastic syndromes (MDS) are clonal haematopoietic stem cell disorders charact-erized by ineffective hematopoiesis, bone marrow (BM) dysplasia, and peripheral cytopenias with a risk of transforming into acute myeloid leukemia (AML) (1). MDS originates from a malignant transformation of a haematopoietic stem cell (HSC), which shows growth advantage over a normal HSC and its clonal expansion (2). The molecular basis of MDS is heterogeneous and the molecular mechanisms underlying pathobiology of this complex disorder is still being understood. About 90% of MDS patients show genetic variations (GVs) in the genes known to be associated with the development of this disorder (3). However, most of the genetic studies on MDS report single nucleotide variations (SNVs) (4, 5) and rarely report insertions and deletions (indels) (6-8). The reported indels in the literature have not been characterized in detail. Nonetheless, investigations have revealed frequent indels in AML (9- 11). Identification of indels in genes that are associated with MDS may provide new insights to understand the molecular basis of the disease to facilitate diagnosis, prognostication, and treatment options. This study investigated the indels of BM derived CD34^+^ haematopoietic stem/progenitor cells (HSPCs) of MDS patients using a targeted next generation sequencing (NGS).

## Materials and methods

**Patients**

Bone marrow samples of newly diagnosed patients (n=20) with *de novo* MDS were collected from four tertiary care hospitals in Sri Lanka (National Hospital of Sri Lanka, Colombo South Teaching Hospital, Colombo North Teaching Hospital and National Cancer Institute, Maharagama) after obtaining the written informed consent. The study was conducted according to the principles of Declaration of Helsinki (2008). Ethical approvals for the study were obtained from the Ethics Review Committee of the Faculty of Medicine, University of Colombo, Sri Lanka and respective hospitals. Patients with secondary MDS were excluded. Patient’s clinical findings and findings of the investigations were recorded. All patients were subtyped according to the WHO classification (12). 

**Isolation and culture expansion of** **CD34** ^+^ **HSPCs**

Mononuclear cells were purified from the BM samples using Histopaque (Sigma-Aldrich, Gillingham, United Kingdom) by density gradient centrifugation and were labeled with CD34 MicroBeads (EasySep CD34 selection kit, Stem Cell Technologies, Canada). CD34^+^ cells were isolated using magnetic cell separation. Flow cytometry (FACScan; Becton Dickinson, Heidelberg, Germany) was used to evaluate the purity of CD34^+^ cell population. Purified CD34^+^ cells were cultured at a concentration of 1.0 × 10^4^ cells/ml in Stemline® Haematopoietic Stem Cell Expansion Medium (SIGMA) supplemented with stem cell factor (50 ng/ml), human Flt-3 ligand (20 ng/ml), thrombopoietin (20 ng/ml), interleukin 6 (50 ng/ml) and antibiotics (100 μg/ml). Cultures were maintained in 25 cm^2^ tissue culture flasks at 37 °C and 5% CO_2_, and cells were expanded for 10 days. 

**Next generation sequencing**

The TruSight Myeloid Sequencing Panel [TMSP (Illumina, San Diego, USA)], which examines mutational hotspots in 54 frequently mutated genes in haematological malignancies, was used to detect variations in the genome of HSPCs. This panel targets 54 genes [15 full genes (exons only) and exonic hotspots of additional 39 genes] (13). DNA sequencing and data analysis were carried out as described previously (14). Briefly, DNA was extracted from HSPCs using QIAamp® DNA Mini kit (Quaigen, USA) and was quantified by “Qubit” fluorometer. DNA libraries were prepared according to the manufacturer’s instructions. Following hybridization of oligos to the target regions, an extension and ligation reaction was performed to combine the oligo pairs across the regions of interest. DNA templates were PCR amplified using index primers. Amplified samples were assessed by running an aliquot of amplified DNA (5 µL) on a 4% agarose gel (amplicon size ≈ 250bp). Finally, libraries were purified using AMPure magnetic beads. The purified libraries were then normalized, quantified and pooled. Pooled libraries with unique barcodes were diluted with chilled HT1 buffer to a final concentration of 12pM and were loaded on a MiSeq Reagent Kit v3 to run on a MiSeq benchtop sequencer (Illumina MiSeq System). Paired end sequencing (2 X 151bp) was conducted according to the default parameters of the Illumina MiSeq System.

**Variant calling and data analysis**

Genetic variants were identified by using two independent bioinformatic pipelines: in-built (Illumina) and in-house. In the in-built method, alignment and variant calling were done using the TruSeq Amplicon App®(Base space®, Illumina) in somatic mode. Somatic variants were also called by an in-house bioinformatic pipeline, MuTect (version 1.1.7) (15). First, the raw sequencing data obtained from the FASTQ were aligned to GrCh37 human genome assembly using Burrows-Wheeler Aligner [BWA (BWA-0.7.12]-mem algorithm. Then the Genome Analysis Tool Kit (GATK-v.3.6) was used to recalibrate the aligned reads. The Binary Alignment Map (BAM) files of MDS patients were co-realigned in pairs with that of a normal healthy Sri Lankan to increase the specificity of MuTect in retaining causal variants. To eliminate population unique variants, a “panel of normals” (PON) was also created by running MuTect on a set of normals. Input arguments (PON and the control BAM files) were run in MuTect on patient samples. The variants common to both pipelines were considered as “True’ variants. Illumina annotation and filtering tool, Variant Studio 3.0® was used to annotate the generated variant caller files. The parameters used in the analysis; filter-pass, quality>30, read depth>250X, minor allele frequency <0.01, alternate allele frequencies >5%, read depth >500X and the alternate allelic depth >20X (16). Catalogue of Somatic Variants in Cancer (COSMIC; http:// cancer. sanger. ac.uk/cosmic) database was used for cross referencing the variants. The analysis was done by two individuals independently. Sanger sequencing was conducted to validate the methodology as described previously (14). In order to predict the potential functional significance, the indels identified by both pipelines were further analyzed with Mutation Taster (www. Mutatio-ntaster.org/).

## Results

**Patient characteristics**

There were 7 males and 13 females in the study population with mean age of 64.5 years (range 31-75 years). Patient’s subtypes, their blood and BM characteristics and karyotypes have been previously published (17).

**Distribution of indels within the genes**

**Fig.1:**
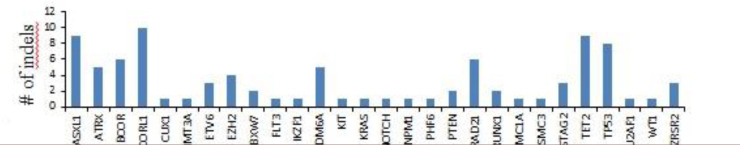
Genes presenting indels in Myelodysplastic syndromes patients

The patient cohort carried 88 indels across 28 genes. These variants included 79 deletions and 9 insertions. HSPCs harbored indels in genes associated with DNA methylation (*DNMT3A *and *TET2*), chromatin remodeling (*ASXL1, ATRX, EZH2, KDM6A, PHF6, FLT3*, and *IKZF1*), transcription regulation (*ETV6, BCOR, BCORL1, RUNX1*, and *CUX1),* cohesion complex (*SMC1A, SMC3, STAG2*, and *RAD21*), spliceosome machinery (*U2AF1 *and *ZRSR2*), signal transduction (*NOTCH1, KRAS*, and *KIT*) and other cellular pathways (*FBXW7, PTEN*, and *NPM1*). The genes that showed more than 5 indels included *BCOR *(N=6), *RAD21 *(N=6), *TP53 *(N=8), *ASXL1 *(N=9), *TET2 *(N=9), and *BCORL1 *(N=10) (Figure 1). Out of these 88 indels, 15 have been previously reported by other research groups (Table 1). The indels per patient in refractory anaemia with excess blasts (RAEB) (N=11) was higher than that of refractory cytopenia with unilineage dysplasia (RCUD) (N=4) and refractory cytopenia with multilineage dysplasia (RCMD) (N=2). 

**Recurrent indels**

Three deletions and two insertions were identified as recurrent indels in our patient cohort (Table 2). The recurrent deletions were W15X in the* EZH2* gene (NM_004456.4: c.43delT), G274X in the *RAD21 *gene (NM_006265.2:c.822delG), and TGAG>TGAG/T in the *BCORL1 *gene (NM_ 021946.4: c.3957_ 3959delGGA). The recurrent insertions were identified in the *FLT3: *E598DYVDFREYE (NM_ 004119.2:c. 1770_ 1793dup CTACGTTGATTTC AGAGAATATGA) and in the *NPM1*: L287LCX (NM_002520. 6:c.860_ 863dupTCTG) genes. Number of patients with recurrent insertions and deletions were: *EZH2 *(N=2), *FLT3, NPM1 *and *RAD21 *(N=3), and *BCORL1 *(N=4). 

**Analysis of downstream effects of indels**

The affected domains of the proteins and the downstream effects are shown in Table 3.

**Table 1:** Previously reported indels

**Gene**	**Variant**	**Type**	**COSMIC ID**	**Primary site**
*ASXL1*	G>G/GC	insertion	110708	haematopoietic and lymphoid tissue
*ASXL1*	AC>AC/A	deletion	307360	haematopoietic and lymphoid tissue
*BCOR*	GT>GT/G	deletion	5453546	large intestine
*DNMT3A*	TC>TC/T	deletion	1583095	haematopoietic and lymphoid tissue
*FBXW7*	CA>CA/C	deletion	5007903	large intestine
*FLT3*	T>T/TTCATATTCTCTGAAATCAACGTAG	insertion	1317912	haematopoietic and lymphoid tissue
*FLT3*	T>T/TTCATATTCTCTGAAATCAACGTAG	insertion	1317912	haematopoietic and lymphoid tissue
*FLT3*	T>T/TTCATATTCTCTGAAATCAACGTAG	insertion	1317912	haematopoietic and lymphoid tissue
*KDM6A*	TA>TA/T	deletion	255005	urinary tract
*NPM1*	C>C/CTCTG	insertion	1319222	haematopoietic and lymphoid tissue
*NPM1*	C>C/CTCTG	insertion	1319222	haematopoietic and lymphoid tissue
*NPM1*	C>C/CTCTG	insertion	1319222	haematopoietic and lymphoid tissue
*PTEN*	AC>AC/A	deletion	125653	endometrium
*PTEN*	TC>TC/T	deletion	5176	Endometrium
*RUNX1*	GC>GC/G	deletion	444420	breast
*TET2*	TA>TA/T	deletion	211709	haematopoietic and lymphoid tissue
*TET2*	TG>TG/T	deletion	120173	haematopoietic and lymphoid tissue
*TET2*	CT>CT/C	deletion	4170105	haematopoietic and lymphoid tissue
*TET2*	TC>TC/T	deletion	87187	haematopoietic and lymphoid tissue

**Table 2:** Recurrent indels

**Gene**	**Variant**	**Amino acid change**	**Type**	**No of patients**
*EZH2 *	CA>CA/C	W15X	deletion	2
*FLT3 *	T>T/TTCATATTCTCTGAAATCAACGTAG	E598DYVDFREYE	insertion	3
*NPM1 *	C>C/CTCTG	L287LCX	insertion	3
*RAD21 *	GC>GC/G	G274X	deletion	3
*BCORL1 *	TGAG>TGAG/T	E1316	deletion	4

**Table 3:** Downstream effects of variants

**Gene**	**Amino acid change **	**Type**	**Affected region/domain & possible effect**
*EZH2 *	W15X	deletion	Interaction with DNMT1, DNMT3A and DNMT3B is lost
*FLT3 *	E598DYVDFREYE	insertion	The region important for normal regulation of the kinase activity is lost.
*NPM1 *	L287LCX	insertion	The region required for nucleolar localization is lost
*RAD21 *	G274X	deletion	D->A: Abolishes cleavage by caspase-3 is lost
*BCORL1 *	E1316	deletion	Nuclear localization signal might get lost

## Discussion

The current study analyzed indels of CD34^+^ HSPCs derived from a cohort of patients with newly diagnosed *de novo* MDS. Deletion in the *BCORL1* at position 1316 was detected as the most recurrent indel (n=4). This was identified as an inframe deletion within the nuclear localization signal motif of BCORL1. BCORL1 is a transcriptional corepressor that binds to class II histone deacetylases (HDAC4, HDAC5, HDAC7), and interacts with the CTBP1 corepressor to regulate the repression of E-cadherin (18). It has been suggested that the loss of function of *BCORL1* may play a role in the progression of MDS to AML (19). The GVs in *BCORL1* have been reported in 5% of the MDS patients (20). Interestingly, in our study cohort this deletion was observed in 20% of patients, thus this could possibly be a molecular marker for the diagnosis of MDS. 

Recurrent deletions were also observed in the *EZH2 *and *RAD21 *genes. EZH2 is an essential component of the polycomb repressive complex 2 (PRC2), which is involved in gene silencing through trimethylation of H3K27 (21). Dysregulation of *EZH2* has been shown to play an oncogenic role in various cancers (22). GVs in *EZH2 *gene are associated with poor prognosis in MDS (23). RAD21 protein is a structural component of the cohesin complex and SNVs in RAD21 have been reported in haematopoietic neoplasms (24). GVs in RAD21 gene are associated with proliferation and differentiation of blood cells (25). Therefore, the detected deletions in RAD21 gene may contribute to the pathogenies of MDS.

Recurrent insertions were observed in the *FLT3* and *NPM1* genes. FLT3 is a class III receptor tyrosine kinase consisting of a juxtamembrane domain (JMD), two tyrosine kinase domains (TKD1 and TKD2), and five extracellular immunoglobulin-like domains (26). GVs of *FLT3* gene have been observed in the internal tandem duplication (ITD) in exon 14 causing the duplication and tandem insertion of the juxtamembrane (JM) domain (27). FLT3 plays a major role in proliferation, differentiation and survival of haematopoietic cells (28). Studies have shown that GVs in internal tandem duplication (ITD) of the FLT3 gene are associated with poor prognosis (29). *NPM1 *encodes for a phospho-protein that belongs to the nucleophosmin/ nucleoplasmin family of proteins, which shuttles between the nucleus and cytoplasm (30). *NPM1 *knocked down mice have shown dysplasia in megakaryocyte and erythrocyte lineages in the BM (31). GVs in *NPM1 *with a prognostic significance have been previously reported in AML (32). Thus, the insertions in the FLT3 and *NPM1 *genes identified in this study may have a clinical importance in MDS pathogenesis, and possess a potential to be used as markers in the diagnosis of MDS.

Our study identified some recurrent indels in MDS which could possibly have a diagnostic, prognostic, and therapeutic importance in MDS. 

However, further investigations with larger cohorts are needed to explore the potential of these indels to be used as biomarkers in MDS.

## Conflict of Interest

The authors declare that they have no conflict of interest
